# The Human Right to Water: The Importance of Domestic *and* Productive Water Rights

**DOI:** 10.1007/s11948-013-9499-3

**Published:** 2013-12-12

**Authors:** Ralph P. Hall, Barbara Van Koppen, Emily Van Houweling

**Affiliations:** 1 School of Public and International Affairs, Urban Affairs and Planning Program, Virginia Tech, 207 Architecture Annex, Blacksburg, VA 24061 USA; 2 International Water Management Institute (IWMI), Pretoria, South Africa; 3 Women and Gender in International Development, Office of International Research, Education, and Development (OIRED), Virginia Tech, Blacksburg, VA 24061 USA

**Keywords:** Human right to water, Multiple-use water services, Domestic-plus, Livelihoods, Gender

## Abstract

The United Nations (UN) Universal Declaration of Human Rights engenders important state commitments to respect, fulfill, and protect a broad range of socio-economic rights. In 2010, a milestone was reached when the UN General Assembly recognized the human right to safe and clean drinking water and sanitation. However, water plays an important role in realizing other human rights such as the right to food and livelihoods, and in realizing the Convention on the Elimination of All Forms of Discrimination against Women. These broader water-related rights have been recognized but have not yet been operationalized. This paper unravels these broader water-related rights in a more holistic interpretation of existing international human rights law. By focusing on an emerging approach to water services provision—known as ‘domestic-plus’ services—the paper argues how this approach operationalizes a comprehensive range of socio-economic rights in rural and peri-urban areas. Domestic-plus services provide water for domestic *and* productive uses around homesteads, which challenges the widespread practice in the public sector of planning and designing water infrastructure for a single-use. Evidence is presented to show that people in rural communities are already using their water supplies planned for domestic uses to support a wide range of productive activities. Domestic-plus services recognize and plan for these multiple-uses, while respecting the priority for clean and safe drinking water. The paper concludes that domestic-plus services operationalize the obligation to progressively fulfill a comprehensive range of indivisible socio-economic rights in rural and peri-urban areas.

## Introduction

Globally, 768 million people lack access to an improved water source, and more than 80 % of these people live in rural areas (WHO and UNICEF 2013). Poor water access is associated with many water-related illnesses, food insecurity, lost productivity, and poor school attendance, especially for women and girls. Daily access to clean water is necessary to satisfy basic needs of drinking, cooking, washing, and bathing—i.e., domestic uses of water. In rural areas, water is also critical for livelihood activities, such as horticulture and crop irrigation, livestock-raising, brick-making, and small-scale commercial activities. These activities increase a household’s income and food security. In peri-urban areas as well, water is necessary for a range of livelihoods (Kurian and McCarney 2010). With rapid urbanization, urban agriculture is becoming particularly important (Zezza and Tasciotti 2010): already in the 1990s, 15–20 % of the world’s food was estimated to be produced in urban areas (Armar-Klemesu 2000).

The formal recognition of access to water as a human right in 2010 was an important milestone in addressing the lack of access to water in developing countries, especially for women. The human right to water was framed from a narrow public health perspective and prioritized the provision of safe and clean water for drinking, sanitation^1^, hygiene, and other domestic activities. Without contesting the priority for domestic uses in human rights law, this interpretation might be seen as ignoring the range of broader socio-economic human rights for which water plays an important role. In general, the operationalization of the human right to water is achieved by providing water services that are planned and designed for domestic uses only. Design norms for service delivery levels typically provide a minimum of 20 litres per capita per day (LPCD), supposedly for domestic activities only. Even in promoting the progressive realization of this right by providing higher service levels, it is assumed that such larger quantities are only used for domestic purposes. In this paper, we trace how the current framing of the human right to safe and clean drinking water as a priority in international law can, and should, go together with the recognition of other water-related human rights, in particular the rights to food, work, and an adequate and continuously improving standard of living. This interpretation accounts for how rural and peri-urban households actually use their water—i.e., for domestic *and* productive uses around the homestead. The paper links these findings to domestic-plus services that offer the opportunity to operationalize state obligations to fulfill the priority for people to access water for domestic uses *and* to realize other human rights.

The paper is structured as follows. Section two provides an overview of the evolution of water as a human right. We argue that the human right to water for domestic uses to meet public health and gender objectives, includes the right to water for livelihoods according to the broader human rights frameworks.

Section three focuses on rural and peri-urban people in low- and middle-income countries and on their norms and practices of water uses, which are not shaped by artificial administrative divides of single-use, sector-based water services provision. People use self-supply and public water schemes for multiple uses, regardless of the use intended by the planners of the scheme. In response to this observation, a new approach of multiple-use water services (MUS) has emerged. Domestic-plus services, which are a form of MUS, prioritize domestic uses at and around homesteads and also promote productive uses, conforming to a range of human rights laws and to the growing recognition that rights are indivisible.

The importance of domestic-plus services is explored in Section four, which presents some results from a multi-country study of household water use from rural piped water systems with and without household connections in Senegal, Kenya, and Colombia. The study offers empirical evidence of the extent (71–75 %) to which rural households engage in both domestic *and* productive uses of water to support their livelihood needs. Further, these activities are supported by low service levels (i.e., water quantities) that are widely seen as suitable for basic domestic uses only.

The paper concludes by summarizing the still untapped potential of domestic-plus services to realize the human right to water for domestic uses and for a range of other water-related socio-economic rights, while recognizing that human rights are indivisible and that people can co-create norms and practices for meeting their human rights.

## The Human Right to Water and Sanitation and Other Water-Related Human Rights

The human right to water and sanitation has evolved from soft to hard international law, where it is now considered as a “distinct, composite human right” (Meier et al. 2012, p. 4). In this section we track the emergence of the human right to water and sanitation, paying particular attention to the way in which the right to water is framed and what this framing implies for rural and peri-urban communities. The discussion reveals how international conventions and comments have been prioritizing the human right to safe and clean drinking water, but not at the exclusion of other uses. An examination of these texts shows a broader interpretation of the right to water without limiting prescriptions on its intended use. States are obliged to protect, respect, and fulfill these other rights too.

In 2010, UN General Assembly recognized “the right to safe and clean drinking water and sanitation as a human right that is essential for the full enjoyment of life and all human rights” (UN 2010b, p. 2). The General Assembly’s Resolution (64/292) brings significant international political weight behind the notion that access to clean and safe drinking water and sanitation is an independent human right.

Access to reliable and clean water and sanitation services is widely accepted as essential for the realization of a healthy and productive life (UNDP 2006; WHO and UNICEF 2011, 2012). Prior to the UN General Assembly’s formal recognition of the right to drinking water and sanitation, access to water and sanitation services was generally considered as a prerequisite for the attainment of other human rights (Gleick 1998). The long road in explicitly recognizing water as a human right has been attributed to a lack of political will and resources in this area when compared to investment in other sectors (UNDP 2006). Since the poor—who suffer the most from a lack of access to improved water and sanitation services—tend to have a limited voice in political arenas, as is often argued, their claims for these services can be more easily ignored if the human right to water and sanitation is not explicit (ibid.). Although progress has been made, this lack of collective action and influence among the poor is one reason for the continuing low level of access to water and sanitation services over the past several decades in developing regions (WHO and UNICEF 2012).

In response to the UN General Assembly’s Resolution (64/292), the UN Human Rights Council (2010, p. 3) called upon states to “develop appropriate tools and mechanisms, which may encompass legislation, comprehensive plans and strategies for the sector, including financial ones, to achieve progressively the full realization of human rights obligations related to access to safe drinking water and sanitation, including in currently unserved and underserved areas.”

Thus, establishing an explicit human right to drinking water and sanitation has created a new mechanism that obliges states to act and target public investments towards the water and sanitation sector.

The following discussion identifies where the focus on drinking water came from and argues that its narrow interpretation can be viewed as necessary to prioritize investments, but that this focus does not ignore other water-related human rights and states’ obligations to meet those rights.

The roots to the 2010 human right to water (for domestic uses) and other water-related rights can be traced to Article 25 of the 1948 Universal Declaration of Human Rights (UDHR) and Articles 11 and 12 of the 1966 International Covenant on Economic, Social, and Cultural Rights (ICESCR). As a group, the UDHR, ICESCR, and the 1966 International Covenant on Civil and Political Rights (ICCPR)—known collectively as the “International Bill of Human Rights”—provide the normative basis from which the human right to water and sanitation and other water-related rights have evolved in international law (Meier et al. 2012; Gupta et al. 2010). These foundational texts also stipulate the obligations for states.

**Universal Declaration of Human Rights (UDHR)**

**Article 25**: “Everyone has the right to a standard of living adequate for the health and well-being of himself and of his family, including food, clothing, housing and medical care and necessary social services,…” (UN 1948).

**International Covenant on Economic, Social and Cultural Rights (ICESCR)**

**Article 2** of the ICESCR calls on states to “individually and through international assistance and co-operation” … work towards “achieving progressively the full realization of the rights recognized in the present Covenant by all appropriate means, including particularly the adoption of legislative measures.”

**Article 11:** “The States Parties to the present Covenant recognize the right of everyone to an adequate standard of living for himself and his family, including adequate food, clothing and housing, and to the continuous improvement of living conditions” (UN 1966b).

**Article 12**: “The States Parties to the present Covenant recognize the right of everyone to the enjoyment of the highest attainable standard of physical and mental health. … The steps to be taken … to achieve the full realization of this right shall include those necessary for … (c) The prevention, treatment and control of epidemic, endemic, occupational and other diseases” (UN 1966b).

**International Covenant on Civil and Political Rights (ICCPR)**

The public health link to water is reinforced by the ICCPR. While the ICCPR does not explicitly mention water, it is considered to be a necessary condition for realizing the “inherent right to life” stated in **Article 6** of the covenant (UN 1966a).

Both the UDHR and ICESCR use the word “including” prior to listing component elements of the right to an adequate standard of living. The word “including” indicates that the elements listed were not meant to be exhaustive, opening the door for the inclusion of other fundamental elements such as water, sanitation, clean air, and food, needed to achieve an adequate living standard (Langford 2005; Gleick 1998). Article 2 of the ICESCR focuses on *all* rights within the Covenant and thus, arguably, includes a right to water for productive purposes derived from an integrated view of the rights to water, food, work, and standard of living.

Article 11 positions water as a necessary element in achieving an “adequate standard of living”. In rural and peri-urban settings, the interpretation of this article needs to be broad. Achieving an adequate standard of living is dependent on the availability of both safe and clean drinking water and on the availability of water to support other domestic and productive uses.

The reference to the “continuous improvement of living conditions” in Article 11 and “progressive realization” in Article 2 of the ICESCR is also worth highlighting. This implies that a priority for providing safe and clean drinking water in rural and peri-urban areas cannot be interpreted as denying families in these locations the opportunity to *continually improve* their circumstances through increasing access to both domestic and productive uses of water. Given the income generating (or expenditure saving), food security, nutritional, and health benefits from productive activities—such as raising animals or growing irrigated crops and vegetables, or aquaculture—an important question is why the right to water has been focused on safe and clean drinking water and what does this imply for other uses? One possible answer lies in Article 12 of the ICESCR, which elevates public health in the human rights dialogue.^2^ The steps taken to attain the highest possible level of physical and mental health include actions that prevent and control diseases, such as the provision of safe and clean drinking water.

One of the first *explicit* references to the human right to water for domestic uses in an international text is found in the conference report from the 1977 United Nations Water Conference in Mar del Plata, which positioned the right to drinking water in the context of basic needs: “all peoples, whatever their stage of development and their social and economic conditions, have the right to have access to drinking water in quantities and of a quality equal to their basic needs” (UN 1977, p. 1). The Mar del Plata conference led to an Action Plan that called for what became the International Drinking Water Supply and Sanitation Decade (1981–1990), which brought international attention and resources to expand access to drinking water supplies and sanitation services in developing regions.

Two years after the Mar del Plata conference, the 1979 Convention on the Elimination of All Forms of Discrimination against Women (CEDAW) made an explicit reference to the rights of women to water. In the context of *rural* *development*, the Convention called for the elimination of discrimination against women and stated that women have the right to “enjoy adequate living conditions, particularly in relation to housing, sanitation, electricity and water supply, transport and communications” (UN 1979, Article 14(2)).

A decade later, the 1989 Convention on the Rights of the Child (CRC) made a clear connection between water and the “highest attainable standard of health” (UN 1989, Article 24(1))—paralleling Article 25 in the UDHR and Article 12 in the ICESCR. To fulfill this right, states are asked, among other measures, to “combat disease and malnutrition … through, inter alia, … the provision of adequate nutritious foods and clean drinking water” (UN 1989, Article 24 (para. 2.c)).

CEDAW and the CRC are particularly relevant to rural and peri-urban communities. These two legally binding treaties establish state obligations and promote the need for monitoring mechanisms to track progress. CEDAW specifically focuses on rural development and references “water supply” in general, rather than focusing only on drinking water and other domestic uses. The CRC explicitly recognizes the right to the “highest attainable standard of health” with nutritious foods and the provision of clean drinking water. While the CRC does not refer to a specific geographic setting, if the rights are considered in a rural and peri-urban context there is a strong case for the operationalization of this right by providing water to grow crops and vegetables, raise poultry, breed livestock, etc. Both CEDAW and the CRC are referenced in Resolution 64/292, and underscore that a priority for domestic uses in operationalizing human rights law still leaves a similar obligation to operationalize rights to water for productive uses.

Turning to the 1990s, the international community began to focus its attention on sustainable development (Ashford and Hall 2011). A year before the Brundtland commission published its classic text on the subject (WCED 1987), the 1986 Declaration on the Right to Development (DRD) was adopted by the UN General Assembly. Article 8 of the Declaration says:

States should undertake … all necessary measures for the realization of the right to development and shall ensure, inter alia, equality of opportunity for all in their access to basic resources, education, health services, food, housing, employment and the fair distribution of income. Effective measures should be undertaken to ensure that women have an active role in the development process … (UN 1986, Article 8).

Within this development framework, the right to water became oriented towards meeting “basic needs”, continuing the operational focus on drinking water as agreed during the 1977 Mar del Plata conference. While the DRD did not explicitly mention water, the UN later included water as a “basic resource” when interpreting the intent of Article 8 (UN 1995; Gleick 1998). Further, the emphasis on including women in the development process, builds on CEDAW. Given women’s and girl-children’s disproportional obligations for water provision for domestic uses, the provision of water services for domestic uses is particularly important to them (UN 2005; Thompson et al. 2001). Moreover, while much attention during the preceding decade was paid to water technology (the “hardware”), an important outcome was that equal attention needed to be paid to the planning and management (the “software”) of the services provided (Cairncross 1992). This *demand responsive* approach to water planning found expression in the 1992 Dublin Statement on Water and Sustainable Development, which considered water as both a social and economic good. Principle 4 of the statement recognizes “the basic right of all human beings to have access to clean water and sanitation at an affordable price” (UN 1992a). The emphasis on water being “affordable” indicates that “the conception of water as an economic good must be limited by the concept of water as a human right in order to ensure equitable distribution of water” (Bluemel 2005, p. 964). Principle 3 of the Dublin Statement also recognized the gender dimension of water, stating that:

**Women play a central part in the provision, management and safeguarding of water**.

This pivotal role of women as providers and users of water and guardians of the living environment has seldom been reflected in institutional arrangements for the development and management of water resources. Acceptance and implementation of this principle requires positive policies to address women’s specific needs and to equip and empower women to participate at all levels in water resources programmes, including decision-making and implementation, in ways defined by them (UN 1992a).

Later that year, the right to development was reaffirmed in the 1992 Rio Declaration. Principle 3 of the Declaration states that “the right to development must be fulfilled so as to equitably meet developmental and environmental needs of present and future generations” (UN 1992b). The action plan for the Rio Declaration—Agenda 21—provided a detailed treatment of the need to protect public health by providing safe drinking water and controlling disease vectors in the aquatic environment. When discussing activities that could be implemented in the area of water supply and sanitation for the unserved rural poor, Agenda 21 asks states to “promote community ownership and rights to water supply and sanitation facilities” (United Nations (UN) 1993, Section 18.76(A) (iv)). Again, in the rural setting, the declaration is about water in general, while its operationalization focuses on safe and clean drinking water.

In 2002, the UN Committee on Economic, Social, and Cultural Rights (CESCR) adopted General Comment No. 15 (GC15), which considers the legal bases of the right to water through Articles 11 and 12 of the ICESCR. (The earlier CESCR General Comments 12 (1999) and 14 (2000) had focused on the right to food and the right to the highest attainable standard of health, respectively, which address the right to an adequate standard of living under Article 11 of the ICESCR.) GC15 determined that the “human right to water is indispensable for leading a life in human dignity. It is a prerequisite for the realization of other human rights” (CESCR 2003, p. 1). GC15 also directly addresses the productive use of water by referencing Article 1 of the ICESCR—which states that “in no case may a people be deprived of its own means of subsistence” (UN 1966b, Article 1, para. 2). The CESCR further states that “parties should ensure that there is adequate access to water for subsistence farming and for securing the livelihoods of indigenous peoples” (CESCR 2003, p. 4). In its discussion, the CESCR makes a distinction between the right to water for personal and domestic uses that support the right to health, and adequate access to water for farming and livelihood activities that support the right to food and the right to work. While the CESCR recognized that water is needed for other purposes such as food production, supporting livelihoods, and cultural practices, it concluded that “priority in the allocation of water must be given to the right to water for personal and domestic uses” (CESCR 2003, p. 3).

The human right to water entitles everyone to sufficient, safe, acceptable, physically accessible and affordable water for personal and domestic uses. An adequate amount of safe water is necessary to prevent death from dehydration, to reduce the risk of water-related disease and to provide for consumption, cooking, personal and domestic hygienic requirements (CESCR 2003, p. 2).

Thus, GC15 prioritizes water for domestic uses, but simultaneously calls for a more inclusive and progressive interpretation of the human right to water for *productive uses* (Hellum 2014). The main components of the GC15 were later reinforced in a report by the United Nations High Commissioner for Human Rights (UNHRC 2007) on the scope and content of the obligations related to the human right to water and sanitation.

In 2004, the UN General Assembly adopted Resolution 58/217 to establish the International Decade for Action, “Water for Life” (2005–2015). The emphasis on “action” was intentional and deemed necessary to achieve the internationally agreed water-related goals contained in Agenda 21, the Programme for the Further Implementation of Agenda 21, the United Nations Millennium Declaration, and the Johannesburg Plan of Implementation (UN 2004). These international texts further advocate the right to development and the right to attain an adequate standard of living, making reference to the International Bill of Human Rights and other key international agreements. In establishing development targets in the Millennium Declaration and the subsequent Millennium Development Goals (MDGs), only water for domestic uses was explicitly mentioned, which resulted in international resources being targeted at this narrower development objective. In fact, the MDGs of reducing by 50 percent the number of people without access to improved water and sanitation, is seen as an arbitrary target by those advocating for the human right to water (UN 2010a; de Albuquerque and Roaf 2012). Instead, Albuquerque and Roaf (2012, pp. 32–33) argue, a “progressive realization” approach should be followed that is based on a continual assessment of national priorities and resource constraints. The ultimate objective is to achieve the full realization of the human right to water and sanitation (i.e., universal access) by adopting a fluid approach to the “obligation to fulfill” the right to water and sanitation.

In 2010, the human right to water was declared in the UN Resolution 64/292. This focused exclusively on the priority for domestic uses as stated in GC15, while adding the right to sanitation. However, as we argued above, GC15, which is the basis for Resolution 64/292, also obligates states to respect, protect, and fulfill other human rights that critically depend on water, including the right to an adequate standard of living, dignity, food, and work.

The priority to provide water for drinking is justified from a public health perspective based on the fact that this is clearly a universal need for everyone, especially children. Moreover, there is political support for implementing the services to meet this need. There is also ample documentation of the *direct* links between safe and clean drinking water and public health improvements. Poor water quality and water shortage is linked to diarrhea, cholera, dysentery, and a variety of other illnesses responsible for high rates of mortality and morbidity in developing countries (Bartram and Cairncross 2010; WHO 2009). Universal access to water for other domestic water needs also contributes to implementing CEDAW because of society’s current gendered division of domestic chores (Hellum 2014).

The question is not about the priority for the human right to water for domestic uses and its progressive realization, but why there is still no operationalization of water for basic *productive* uses, let alone any effort for its progressive realization, in spite of GC15. Various factors play a role. Water is just one input in productive activities, so benefits are more indirect. Nevertheless, these benefits are important. For example, if water is used for horticulture or animal raising, the products from these activities improve the nutrition of family members and, hence, their health. They often also generate an income that can be used to purchase clothes or send children to school. These various benefits represent a composite group of rights, which fail to match the administrative categories of specific rights. Instead, these multiple interconnected benefits of water for domestic and a range of productive uses underline the importance of recognizing human rights as indivisible (Hellum 2014). Moreover, productive water uses vary and depend on highly diverse hydrological, technical, institutional, and socio-economic contexts. Uptake of water for productive uses depends on factors such as access to land, credit, and markets, and the level of income and education achieved by household members (Van Houweling et al. 2012; Hall et al. 2011). While the diversified livelihood strategies in rural and peri-urban areas depend in many ways on water, the universality of water for productive needs is less straightforward than for drinking. Further, there is less political support and funding for pro-poor agricultural water management.

Lastly, and the focus of the remainder of this paper, water use norms and practices tend to be overlooked in developing and operationalizing human rights law. While the indivisibility of water-related rights is recognized at abstract levels, this tends to be ignored in designing interventions to meet human rights. The following two sections look at how people in rural and peri-urban areas use water and how designing water services to match these uses might advance the realization of water-related human rights.

## People’s Multiple Water Uses in Rural and Peri-Urban Settings

In the past decade, water professionals’ understanding of how rural and peri-urban people use water has been evolving beyond the fragmented disciplinary backgrounds and organization of the water sector that focuses on single water uses. In the early 1970s, the groundbreaking study on domestic water use in East Africa, entitled *Drawers of Water*, by White et al. (1972) developed three categories of water use—*consumptive* (drinking and cooking), *hygiene* (washing, cleaning, and bathing), and *amenities* (watering lawns and other non-essential activities). In a follow-up study some 30 years later, Thompson et al. (2001) added *productive uses* as a fourth category. Productive uses were considered to include “consumption by livestock (e.g., cattle, goats, pigs and sheep), brewing beer, distilling gin, making fruit juice, brick-making and the construction of homes, and irrigating tree and horticultural crops” (Thompson et al. 2001, p. 31).

*Drawers of Water II* demonstrated that the productive use of water intended for domestic uses only by rural households (from piped and non-piped sources) was a largely unrecognized, but important factor supporting livelihoods.

What is interesting is the significant quantities [of water] used by rural households, particularly those with piped supplies. This suggests that access to piped water is beneficial to rural households from a productive as well as a health and well-being perspective (Thompson et al. 2001, p. 31).

Others in the domestic sector also recognized the productive use of water around the homestead from water supplies planned for domestic uses, and the impact these activities have on reducing poverty, empowering women, and improving the sustainability of water services (Moriarty and Butterworth 2003; Moriarty et al. 2004; Torres et al. 2003; Thompson et al. 2001; van Koppen et al. 2006; World Bank, FOA and IFAD 2009). The same observations were made where schemes designed for irrigation were used for domestic uses and non-irrigation productive uses in the area around the homestead (Bakker et al. 1999; Li et al. 2005; Meinzen-Dick and van der Hoek 2001; Renwick 2001; Smith 2004).

Water projects typically supply water for a single-use—such as for domestic use or irrigation—while people use water for all their needs. Regulations or policies often try to forbid non-planned uses, or even declare these uses as illegal. It is true that such non-planned uses may cause damage, for example, when cattle trample ditches. On the other hand, these non-planned uses reflect people’s norms and priorities and meet basic livelihood needs. They realize a range of human rights.

Instead of forbidding non-planned uses, such uses should be planned for. This notion led to the new approach of MUS as a *whole water approach* that responds to the many water needs of rural and peri-urban households (de Vries et al. 2004; Penning de Vries 2006; Renwick et al. 2007; Smits et al. 2010; van Koppen et al. 2006; Restrepo Tarquino 2010; World Bank et al. 2009).

MUS is defined as “a participatory, integrated and poverty-reduction focused approach in poor rural and peri-urban areas, which takes people’s multiple water needs as a starting point for providing integrated services, moving beyond the conventional sectoral barriers of the domestic and productive [irrigation] sectors” (van Koppen et al. 2006, p. v). The participatory (ground-up) nature of MUS means that the services respond to the full range of water needs, including productive activities such as agriculture, gardening, horticulture, livestock-raising, car-washing, arts, ice-making, brick-making, pottery, butchery, and other small-scale commercial activities (van Koppen et al. 2009; Smits et al. 2010). Water-dependent activities provide critical income streams, especially for the rural poor who often lack opportunities for wage and salary work (Smits et al. 2010; Noel et al. 2010). The concept of MUS holds much potential, since between 60 and 70 % of the rural poor are estimated to raise livestock, have access to small cultivable plots, and engage in water-dependent small enterprises (Renwick et al. 2007).

Three service categories have emerged in the past decade that explicitly recognize the need to provide water for domestic and productive uses.^3^ First, in a “MUS-by-design” service, the multiple water needs of all community members are considered according to their prioritization in the design of the system(s). MUS-by-design is an *approach* that tries to match the available water sources with the users’ needs in a participatory way. Thus, there is no one blueprint that can be followed. An MUS-by-design water service could consist of one standalone system or several systems that utilize multiple sources supporting various uses.

The second services category is “irrigation-plus.” An irrigation-plus service prioritizes water for irrigation, but also includes add-on system components that enable the communities in the irrigated area or downstream to use the water for domestic and other productive activities.

The third category is “domestic-plus” services. Domestic-plus services prioritize domestic water uses at or around homesteads and provide services to meet other water-related basic needs. By increasing the design flow of the system (or service levels), greater quantities of water are provided to homesteads to enable more uses. Domestic-plus is progressively implemented by ‘climbing the multiple use water ladder’ (Renwick et al. 2007). Further, components can be added such as cattle troughs and water tanks to support productive activities. Thus, the domestic-plus approach aligns with the priority for domestic uses embedded in the current interpretation of the human right to water and simultaneously meets other water-related needs and human rights. Moreover, domestic-plus services can especially benefit women, the landless, and the sick, for whom homesteads are the best or only place to use water productively. The incremental investment costs to move to such higher service levels, for example, from 20 to 50 LPCD, generate high incremental benefits. Renwick et al. (2007) calculated that such incremental costs could be fully repaid within 6 months to 3 years from the income gained from the productive activities.

Domestic-plus recognizes the importance of health benefits from safe and clean drinking water—that remains safe and clean at the point of consumption. However, the focus is on 5–10 LPCD for drinking, cooking, and personal hygiene. For the larger quantities to support other domestic and productive activities, non-potable water could be used, also saving costs. Point of use (POU) technologies can be used to ensure the 5–10 LPCD is fit for consumption. Evidence on the link between water quality interventions (in general) and health, reveals that a 42 % reduction in child diarrhea morbidity can be achieved (Waddington et al. 2009). For POU treatment interventions, this figure increases to 46 %, but declines to 21 % for interventions that only improve the quality of the water source (ibid.).

Prior to promoting a widespread scaling of domestic-plus services as a cost-effective way to meet a range of human rights, the extent to which domestic water supplies are used for productive activities needs to be understood. The following empirical study of household water use from rural piped water systems in Senegal, Kenya, and Colombia provides some evidence of the extent to which domestic water is repurposed in rural communities across these three countries.

## The Productive Use of Rural Water Supplies Designed for Domestic Uses: Evidence from Senegal, Kenya, and Colombia

Given the growing interest in MUS, the Water and Sanitation Program (WSP), World Bank, funded a multi-country study to assess the link between the productive use of piped, rural domestic water systems, poverty-reduction, and system sustainability. The study—undertaken during the summers of 2008 and 2009 under the direction of the lead author and two colleagues at Stanford University and the University of Oxford—used a cross-sectional research design and focused on rural communities in Senegal, Kenya, and Columbia.^4^ A typical water system in Colombia used mountain spring sources and had a gravity fed distribution system. In Kenya, a mix of gravity and pumped systems supplied communities. In Senegal, all the systems used groundwater that was pumped to an elevated storage tank and then piped to communal taps and a limited number of yard/compound connections. The systems studied in Senegal can be classified as providing domestic-plus services, whereas the systems in Kenya and Colombia were primarily designed as single-use, domestic water services.

The study revealed that a high proportion of households were engaged in productive uses of water (see Table 1). In the three countries, between 71 and 75 % of all households interviewed were engaged in one or more productive activities that used any water source. Piped water supplies were a more important source than other water sources: between 54 and 61 % of households used piped water to support their activities. These uses met various needs: between 34 and 43 % of the households generated an income from their piped-water-based activities. When considering productive activities supported by any water source, between 49 and 55 % of households reported generating an income from their activities. Based on these descriptive data, the potential income from the productive use of water may provide sufficient financial resources to repay the incremental improvement in water supply from domestic services only to domestic-plus water services.

**Table 1:** Household engagement in productive activities in Senegal, Kenya, and Colombia

Item	Senegal (domestic-plus services) (*n* = 1,860)	Kenya (domestic services) (*n* = 1,916)	Colombia (domestic services) (*n* = 1,819)
Median water consumption (LPDC)	23	31	133
Average number of people per HH	13	5.1	3.6
Percentage of HHs that were engaged in one or more productive activities	97	96	84
Percentage of HHs that were engaged in one or more productive activities that used any source of water	74	71	75
Percentage of HHs that were engaged in one or more productive activities that used piped water	54	54	61
Percentage of HHs that earned an income from their piped-water-based activities	34	43	39
Percentage of HHs that earned an income from their water-based activities (using piped and non-piped water)	49	55	51

Given the variation that exists in the types of piped water systems studied, the similarity in proportions of households engaged in piped-water-based productive activities in the three countries is even more remarkable. It is also interesting that the systems in Senegal, which were designed to provide water for livestock and small-scale agriculture, and can thus be classified as domestic-plus services, had the same proportion of households engaged in productive activities as in Kenya and Colombia, where the systems were designed for domestic uses only. This consistent pattern implies that rural households use their water as needed and do not limit their engagement in activities due to limitations in the design of services. Further research is required to determine whether the pattern of engagement in productive activities across the three countries is a more universal phenomenon, or is limited to the purposefully-sampled piped systems included in the study.

We note that the amount of water used, the types of activities undertaken, and the amount of income generated from productive activities varies significantly (between countries and between different regions within countries). For example, in Senegal, the regions of St. Louis and Matam can be characterized as the dominant livestock regions, whereas agriculture is more important in Diourbel and Kaffrine. Further, the median water use per capita in Colombia was several times that of the African households. This high level of piped water consumption can be attributed to the fact that virtually all of the households interviewed in Colombia had access to a private tap. In contrast, only around one third of households in Senegal and Kenya had a comparable level of service. In these countries, far more households relied on public taps or a neighbor’s tap.

The data in Table 1 highlight the fact that while there may be a consistent pattern for household engagement in piped-water-related productive activities, the quantity of water used for these activities is dependent on the level of water access and availability. With regards to Senegal and Kenya, households use a median of 23 and 31 LPCD, respectively. This is in sharp contrast to the general global consensus that around 50 LPCD is required to satisfy an individual’s personal and domestic water needs, with 20 LPCD set as a minimum for consumption and hygiene requirements (Gleick 1996; Langford 2005; Howard and Bartram 2003). The WSP study found that even at these minimum levels, people prioritize water uses for a wide range of livelihood-related activities over domestic uses. This finding reinforces the argument that human rights law and water services planning should better align with people’s priorities.

While homestead-based production is generally considered to consist of a ‘kitchen garden’ for family consumption, Table 1 sheds new light on the importance of the income generated. In Senegal, where the systems were designed to support both domestic and productive uses of water, water-based income represented around one half of total household income (for the 49 % of households engaged in water-based income generating activities). Similarly, in Colombia, income from water-based activities represented, on average, 52 % of total household income (for the 51 % of households engaged in water-based income generating activities).

The income generated was particularly relevant for women. Evidence from the women’s focus groups in Senegal shows that after the construction of the domestic-plus water services, women were able to expand their existing livelihood activities and initiate new enterprises. One half of women’s income was linked to productive water use, namely through livestock-raising and gardening (Van Houweling et al. 2012). This confirms that water provided for productive uses gives women opportunities to diversify their livelihood activities, and earn income from activities that they tend to have greater control over (Van Houweling et al. 2012; van Koppen et al. 2009).

The WSP study shows that productive water use occurs across a range of piped water systems and the types of productive activities vary by context. This finding implies that expanding the human right to water should not target (or exclude) certain uses or technologies, and should, instead, explicitly address the right to water to meet the full range of domestic, health, sanitation, and livelihood needs.

## Conclusion

An important question underlying this paper is whether the current formulation of the human right to safe and clean drinking water (in UN Resolution 64/292), could limit development opportunities for rural and peri-urban communities. Emerging evidence shows that a significant proportion of households in rural and peri-urban communities use water for both domestic *and* productive activities, regardless of whether these dual uses were considered in the design of their water services.

The UN General Assembly Resolution is likely to lead to “enhanced opportunities for rights-based water and sanitation policy” (Meier et al. 2012, p. 3). The key issue is what types of water interventions will be implemented if the focus remains solely on safe and clean drinking water. The UN Human Rights Council (2010, p. 3) has already called on states to develop a broad range of approaches “to achieve progressively the full realization of human rights obligations related to access to safe drinking water and sanitation, including in currently unserved and underserved areas”. Here, we have argued that, within the broad range of possible approaches to translate this state obligation into actual interventions, domestic-plus services address the priority right of water for domestic uses, and also enable other human rights such as the right to food, work, and an adequate standard of living.

When water is provided to meet both domestic *and* productive water needs, a wide range of benefits can be realized. The domestic-plus approach has been associated with multiple poverty impacts, including income, food security/nutrition, health, gender equity, and reduced vulnerability and livelihood diversification. These impacts align well with Article 12 of the ICESCR, which states that everyone has the right to “the continuous improvement of living conditions”. If water service providers interpret the priority for safe and clean drinking water at the exclusion of meeting other human rights, the potential benefits from productive activities may be undermined, contradicting the intent of Article 12.

There are two potential outcomes from a failure to account for the productive use of water in the design of rural and peri-urban services: (1) a higher than expected water demand may prevent the services from reaching all of the intended users, and cause unplanned stress on the water system, potentially undermining the technical and financial performance of the services, and hence, their long-term sustainability; and (2) the households may have no choice but to reorient their domestic and productive uses of water so they live within the service design parameters. In the latter scenario, the water services may operate for their design life, but the potential development opportunities for households could be constrained. The results from the WSP study show that the percentage of households engaged in the productive use of water was relatively consistent across a wide range of water consumption—i.e., from 23 LPCD in Senegal to 133 LPCD in Colombia. These findings challenge the notion that the way in which water is used is constrained by its availability. While the international standard of 50 LPCD is a valuable target, an equally important factor in rural and peri-urban areas is that domestic *and* productive uses of water are taken into account in the design of a water service to maximize health and economic benefits. Attention should also be paid to the gendered nature of water to ensure that the benefits from productive water use accrue to women as well as men.

The current focus on the human right to safe and clean drinking water, does not explicitly address the gendered nature of water collection and use. The Dublin statement, CEDAW, and the DRD explicitly recognize the importance of including women in the design and management of water services to ensure that the services respond to their basic needs, however, most water planning approaches do not involve women, and may therefore marginalize their needs. The domestic-plus approach, strives to provide a new model that considers the full range of needs of women and other vulnerable groups. Following this approach, systems are creatively designed to facilitate women’s water-based domestic activities, as well as their productive activities.

While a water system may be designed to support productive uses of water, not all households will engage in such activities. Around one quarter of all households in Senegal, Kenya, and Colombia did not undertake any productive activities that relied on water. These data indicate that the right to water for productive uses is perhaps less universal that the right to water for domestic uses. However, given the potential benefits that can be realized from productive uses of water, all households should have the opportunity to engage in such activities.

The main concern expressed throughout this paper is that the current focus on the human right to safe and clean drinking water may limit the impact of water service provision if it focuses solely on domestic water supply. However, the human rights framework is one of several factors that shape investment in water services. For example, the Millennium Development Goal to “halve, by 2015, the proportion of people without sustainable access to safe drinking water and basic sanitation”, has led to a significant global investment in enhancing access to improved water sources, but does not recognize the need for water for productive uses. Thus, reorienting the human right to water to incorporate a more holistic concept of water use is one critical step forward. But international development initiatives (such as the MDGs and their successors) also need to be reframed to consider the importance of water for food, livelihood, and standard of living.

Water is vital to livelihoods and to the prospects of rural and peri-urban residents escaping poverty. The human right to water is fundamental to the right to life, health, and food, and as the Vienna Declaration of Human Rights proclaimed “human rights are universal, indivisible and interdependent and interrelated.” We conclude that the human right to water should not be limited to safe and clean drinking water and a more progressive interpretation of existing international law, focusing on the human right to water (in general), may be a more effective way to address a comprehensive range of socio-economic rights in rural and peri-urban areas. This more open interpretation also provides states with more options for the provision of water services when taking concrete actions to respect, fulfill, and protect the human right to water. We present the domestic-plus, irrigation-plus, and MUS approaches to the delivery of water services as important options to help progressively realize a broad range of water-related human rights.

## Footnotes

1 While we recognize the urgent need to realize the human right to sanitation services, this paper focuses on the need to include productive water uses in the formulation of the human right to water. This more holistic approach implicitly supports the delivery of sanitation services. For example, eco-sanitation services could provide valuable nutrients for small-scale horticulture activities supported by a domestic-plus water service, and adequate sanitation is necessary to achieve the desired health benefits of improved water systems. For an explicit discussion on the right to sanitation and hygiene, see de Albuquerque (2009).

2 The public health link to water is reinforced by the ICCPR.

3 These groups were agreed upon during an MUS Roundtable Workshop held at the Rockefeller Bellagio Center in Italy, September 3–7, 2012. The workshop was attended by nineteen participants from around the world representing government, non-governmental organizations, and academia who are active in the implementation or evaluation of MUS. The first two authors of this paper attended this workshop.

4 Within each country, a purposive sampling strategy was used to select around 50 communities with piped water systems (147 systems were studied in total). The sample was designed to capture some variation in the extent of household-based productive activities undertaken in the communities, such that ‘similar’ communities with different levels of productive activity could be compared. Given this approach, the results cannot be considered as representative at the national level in each country. The data collection instruments included household surveys; an engineering assessment of installed infrastructure; interviews with community leaders, water committees, and water system operators; and focus groups with women (and men in Colombia).
